# Evaluation of parental knowledge after establishing CAH clubs in Vietnam & Indonesia

**DOI:** 10.1186/1687-9856-2015-S1-P53

**Published:** 2015-04-28

**Authors:** Irene Mitchelhill, Kate Armstrong, Maria Craig, Vu Chi Dung, Bui Phuong Thao, Nguyen Ngoc Khanh, Thi Bich Ngoc, Thuy Thi Diem Hoang, Huynh Quynh, Dong Trieu Phuong Tran, Pham Ngoc Trach, Huynh Thoai Loan, Nguyen Phuong Khanh, Tran Thi Bich Huyen, Aman Pulungan, Frida Soesanti

**Affiliations:** 1 Department of Endocrinology, Sydney Children’s Hospital (SCHN), Randwick, NSW, Australia; 2 CLAN: Caring Living with Neighbours, Eastwood, NSW, Australia; 3 Department of Endocrinology, Children’s Hospital (SCHN) Westmead, Sydney, NSW, Australia; 4 Department of Endocrinology, National Hospital of Pediatrics, Hanoi, Vietnam; 5 Department of Nephrology-Endocrinology, Children's Hospital 2, Ho Chi Minh City, Vietnam; 6 Department of Pediatrics, Faculty of Medicine, University of Medicine, Ho Chi Minh City, Vietnam; 7 Department of Nephrology-Endocrinology, Children’s Hospital 1, Ho Chi Minh City, Vietnam; 8 Department of Child Health, Faculty of Medicine, University of Indonesia, Jakarta, Indonesia

## 

The incidence of Congenital Adrenal Hyperplasia (CAH) in some Asian countries is far higher than in Australia, (eg 1:6000 as per the Filipino Newborn Screening Program). For many families in low and middle-income countries in Asia resources are limited, affordable and reliable access to essential medicines is problematic, and families living remotely are required to travel long distances for medical care [1]. CAH is associated with significant physical & psychosocial consequences for affected children & their families where treatment is suboptimal, so there are important equity implications for the global CAH and paediatric endocrinology communities to consider.

Health education is an integral component of health care in any setting. “CAHPepTalk” is an educational resource that was developed initially for CAH families in Australia. This validated educational resource has been produced in DVD format and can be facilitated by one health professional. The program includes a translated validated CAH Knowledge Assessment Questionnaire (CAHKAQ) [2], to enable staff to assess patient knowledge in order to evaluate educational needs.

In collaboration with CLAN (Caring & Living As Neighbours), an Australian NGO committed to optimal quality of life for all children living with chronic health conditions), translation of “CAHPepTalk” into Vietnamese & Indonesian was undertaken, & distributed to CAH Communities in the Asia-Pacific region, was supported by CLAN.

Knowledge of parents of children with CAH in Vietnam and Indonesia was assessed prior to families attending education programs run at CAH Club meetings supported by CLAN, in 3 settings: Hanoi, Ho Chi Minh City & Jakarta. 260 questionnaires have been completed by parents. The results to be presented will include knowledge, management & demographics.

Using the CAHKAQ is the first step in the education process in order to improve health outcomes for families in any setting, within Australia, Vietnam or Indonesia.
